# Port Site Obstructed Hernia in a Morbidly Obese Patient: A Case Report

**DOI:** 10.7759/cureus.42264

**Published:** 2023-07-21

**Authors:** Kumar Vineet, Shweta Rai, Vibha Mishra

**Affiliations:** 1 Surgical Oncology, Mahamana Pandit Madan Mohan Malaviya Cancer Centre (MPMMCC) & Homi Bhabha Cancer Hospital (HBCH), Varanasi, IND; 2 Gynecologic Oncology, Mahamana Pandit Madan Mohan Malaviya Cancer Centre (MPMMCC) & Homi Bhabha Cancer Hospital (HBCH), Varanasi, IND

**Keywords:** port site hernia, obesity, laparoscopic surgery, obstruction, trocar site hernia

## Abstract

Indications for laparoscopic surgeries are increasing in the current era in view of the advantages they offer in terms of less perioperative morbidities, early mobilization, and better cosmesis. These benefits are perceived even more in obese women. However, there are special challenges in this population, associated with their body habitus, poor visibility, and perioperative anesthesia risks. Difficulty in port closure is one such problem encountered in these women causing inadequate rectus suturing and leading to port site hernia. We report a case of a 59-year-old morbidly obese lady who underwent a total laparoscopic hysterectomy, bilateral salpingo-oophorectomy, and pelvic lymph node dissection for carcinoma endometrium. The intraoperative course was uneventful. In the postoperative period, she developed acute obstruction due to port site herniation of the small bowel, which was not suspected till postoperative day five. This was due to an inaccurate assessment of her abdomen because of her body habitus. A CT scan was done in view of the non-resolving obstruction, which revealed herniation of a small bowel loop through the umbilical port. Immediate correction was resorted to under local anesthesia. Rectus sheath closure was done in the same sitting. The patient had a quick recovery after that and was discharged three days later. Rectus sheath closure should be done for all ports 10 mm or greater in diameter. There should be a low threshold to get cross-sectional imaging in postoperative obese women with non-resolving gastrointestinal symptoms.

## Introduction

Laparoscopic surgery has special advantages in morbidly obese women in terms of early postoperative mobilization and recovery and less wound infection [1]. However, these women pose unique problems to the treating team in terms of gaining access to the operating site and intraoperative visibility issues [2]. Perioperative anesthesia concerns are aggravated due to difficult intubation, ventilation problems leading to increased peak pressure, and the steep head-low needed in these cases [3].

Also, postoperatively, the closure of the rectus sheath in ports is a problem even with all available expertise and port closure instruments. This generally leads to inadequate closure, which causes hernia formation at the port site, which is quite often ignored due to late presentation.

Here, we report one such case where inadequate closure of the rectus sheath led to strangulated hernia in the immediate postoperative period.

## Case presentation

**Figure 1:**
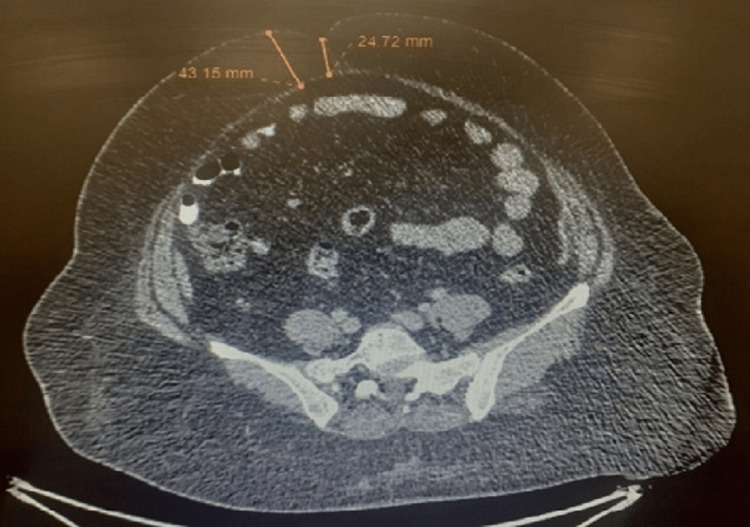
Cross-sectional cut of the preoperative CT scan at the level of the umbilicus showing a 4.15 cm depth of subcutaneous fat layer in the anterior abdominal wall

A 59-year-old post-menopausal lady presented to the outpatient department with complaints of postmenopausal bleeding. Further evaluation with imaging and endometrial biopsy revealed the diagnosis of carcinoma endometrium limited to the uterus. She was also a known case of diabetes mellitus with a body mass index of 45.94 kg/m^2^. After clearance from anesthesia and a complete preoperative workup, she underwent a total laparoscopic hysterectomy, bilateral salpingo-oophorectomy, and pelvic lymph node dissection. The intraoperative course was uneventful but her 12 mm umbilical port sheath closure could not be ascertained due to the sheer volume of subcutaneous fat (Figure 1), and it was decided to also apply subcutaneous sutures after the sheath closure sutures.

**Figure 2:**
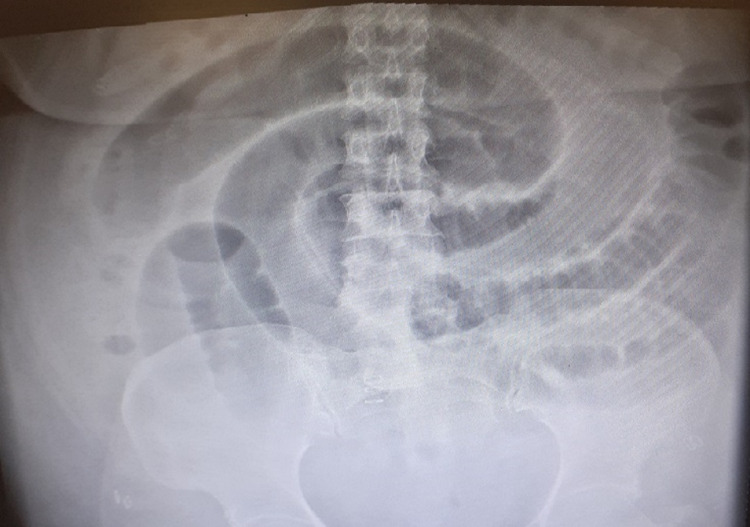
POD 4 X-ray of the abdomen showing dilated small bowel loops POD: postoperative day

She was allowed clear liquids on the same day of surgery. She complained of nausea from postoperative day (POD) two, but it was contemplated that recovery may be slow due to her co-morbidities. On POD four, after allowing a liquid diet, she had multiple episodes of vomiting. Again, there were no signs of abdominal discomfort like tenderness or guarding and no significant distension of the abdomen. A supine abdominal X-ray was sought (Figure 2) which revealed dilated small bowel loops. A Ryles tube was inserted, which had significant output. The patient was made nil by mouth and conservative management continued.

**Figure 3:**
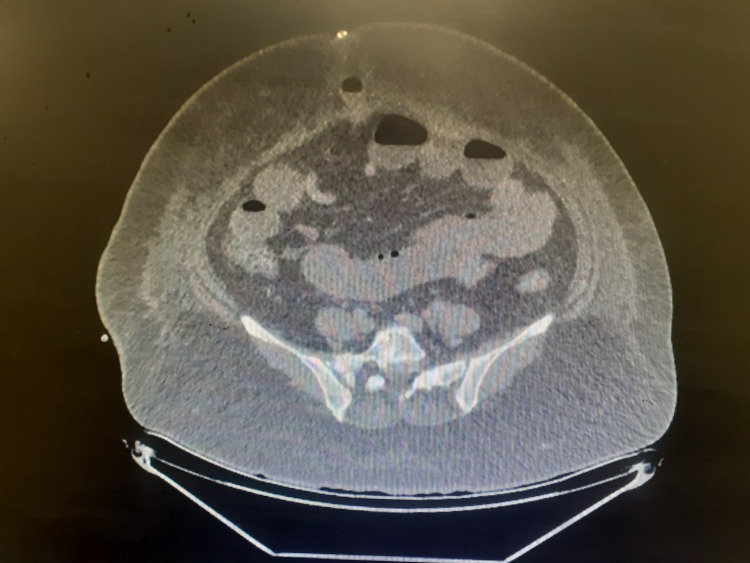
Cross-sectional cut of the CT scan at the level of the umbilicus showing the herniated small bowel loop in the umbilical port

The obstruction was not resolving till POD 7 though she still had no signs of hemodynamic instability. Computed tomography (CT) scan of the abdomen and pelvis was done, which revealed herniation of a small bowel loop in the umbilical port (Figure 3).

In view of her morbid obesity with associated anesthesia risks, it was decided to go for wound exploration under 2% lignocaine local anesthesia, with an intent to resort to exploration under general anesthesia in the same sitting in case the attempt failed. Due to the sheer thickness of the anterior abdominal wall, it was not possible to visualize the involved intestinal segment, so we used a 5 mm laparoscope to visualize the obstructed bowel in the abdominal wall by keeping it about 1 cm inside the umbilical port. A viable-appearing small bowel loop was seen herniating through the sheath with a narrow neck. Gentle digital reduction of the loop was done by releasing the loops from the rectus sheath margins. The length of the umbilical port incision on the skin was increased by around 1.5 cm (in total 2.8 cm) and thereafter, the sheath was closed properly with polyglactin suture and skin clipped. The patient had bowel movements on the same day of the procedure. The gradual escalation of diet was done considering the possibility of an ischemic bowel segment. She tolerated the diet well and was discharged on POD 12 uneventfully.

## Discussion

Port site hernia (PSH) or trocar site hernia (TSH) is one of the common complications of laparoscopic surgery. The reported incidence ranges from 0.65% to 2.8% [4-5]. In a recent single-center study of gynecological procedures, its incidence was found to be 0.016% [6]. Further, in a survey done by the American Association of Gynaecologic Laparoscopists, its incidence was calculated to be 21 per 100,000 [7]. The rate of PSH is significantly higher in the older population than in younger ones [6].

Tonouchi et al. tried to classify PSH into three types - early-onset, late-onset, and special type. Early-onset PSH occurs due to dehiscence of fascial planes and peritoneum within two weeks and presents most commonly with small bowel obstruction. The late type occurs after two weeks and usually has a dehiscence of the fascial plane with a sac consisting of a peritoneum. About two-thirds of cases of PSH are late-onset type. Since most patients are asymptomatic, calculating the true value is difficult [8]. Around 12% of late-onset PSH presents with obstruction. Thus, the incidence of PSH calculated in various literature is likely to be misleading in the sense that they mostly represent the incidence of early-onset PSH and symptomatic PSH.

There are many risk factors associated with the development of port site hernia such as obesity, female gender, and the use of a 10 mm trocar at midline [9]. All these risk factors were present in our patient. This was a case of an early-onset port site strangulated hernia. We initially tried to manage the case conservatively since our initial differential with limited abdominal findings was postoperative ileus. Plain radiography in the form of X-ray is nonspecific and generally non-contributory to the cause of obstruction. In our case, it only showed dilated central bowel loop in an inverted S fashion. An abdominal CT scan was resorted to since the obstruction was not resolving to conservative treatment. This modality is effective in diagnosing the site and cause of obstruction, and in this case, it showed strangulated jejunal loop at the port site [10].

In morbidly obese patients, their body habitus causes masking of symptoms and abdominal findings of strangulated hernias. This may lead to late recognition of obstruction and catastrophic results in the form of ischemia, gangrene, or perforation of the bowel segment. Therefore, the threshold for performing an abdominal CT scan should be low, especially if the obstruction is not resolving with conservative treatment.

The risk factors associated with the surgical technique, which increase the risk of PSH are the widening of the fascial defect by the use of ‘fascial screws’, large ports, cutting trocars, and inadequate closure of larger fascial defects (>7 mm) [11]. In 2016, Singal et al. in their prospective study of 200 non-obese patients concluded that closure of 10 mm or greater ports does not reduce port site hernia. However, the same may not be true for obese women [12]. In our case, the main cause of the port site hernia was not ascertaining whether the closure of the port site rectus sheath is adequate. The dilemma to increase the incision length after the completion of the laparoscopic operation led to a near-miss major postoperative morbidity in this woman.

Therefore, meticulous closure of port site fascial defects should be a priority for these women. There are many special materials available for aiding in fascial defect closure, like port site suture with a thick needle and curve, laparoscopic port closure system, which generally includes closure forceps, the port closure 2 mm forceps model, port closure Mochi type, and knot pusher, etc. In the absence of these techniques, one should not hesitate to increase the skin incision for a few centimeters to access the rectus sheath well for proper suturing.

## Conclusions

Our experience from this case leads us to the conclusion that all port site sheaths/fascia > 10 mm should be closed adequately even if it requires an increasing length of port site incision. Also, there should be a low threshold to get cross-sectional imaging in unsettled obese women.
